# A Design Review for Biomedical Wireless Power Transfer Systems with a Three-Coil Inductive Link through a Case Study for NICU Applications

**DOI:** 10.3390/electronics13193947

**Published:** 2024-10-07

**Authors:** Amin Hazrati Marangalou, Miguel Gonzalez, Nathaniel Reppucci, Ulkuhan Guler

**Affiliations:** Department of Electrical and Computer Engineering, Worcester Polytechnic Institute, Worcester, MA 01609, USA

**Keywords:** wireless power transfer, power delivered to load, wearable biomedical devices, class-E power amplifier, energy harvesting, inductive coupling power transfer, power transfer efficiency, neonatal intensive care, mattress

## Abstract

This paper outlines a design approach for biomedical wireless power transfer systems with a focus on three-coil inductive links for neonatal intensive care unit applications. The relevant literature has been explored to support the design approach, equations, simulation results, and the process of experimental analysis. The paper begins with a brief overview of various power amplifier classes, followed by an in-depth examination of the most common power amplifiers used in biomedical wireless power transfer systems. Among the traditional linear and switching amplifier classes, class-D and class-E switching amplifiers are highlighted for their enhanced efficiency and straightforward implementation in biomedical contexts. The impact of load variation on these systems is also discussed. This paper then explores the basic concepts and essential equations governing inductive links, comparing two-coil and multi-coil configurations. In the following, the paper discusses foundational coil parameters and provides theoretical and experimental analysis of both two-coil and multi-coil inductive links through step-by-step measurement techniques using lab equipment and addressing the relevant challenges. Finally, a case study for neonatal intensive care unit applications is presented, showcasing a wireless power transfer system operating at 13.56 MHz for powering a wearable device on a patient lying on a mattress. An inductive link with a transmitter coil embedded in a mattress is designed to supply power to a load at distances ranging from 4 cm to 12 cm, simulating the mattress-to-chest distance of an infant. the experimental results of a three-coil inductive link equipped with a Class-E power amplifier are reported, demonstrating power transfer efficiency ranging from 75% to 25% and power delivery to a 500 Ω-load varying from 340 mW to 25 mW over various distances.

## 1. Introduction

Wireless power transfer (WPT) has generated significant interest across various domains due to its ability to deliver power across a broad spectrum, ranging from nanowatts in certain Internet of Things (IoT) applications [1] to milliwatts in wearable and implantable medical devices [2–4], to watts in assisted living or industrial applications [5,6], and even kilowatts in electric vehicles (EV) [7]. Inductive coupling is a prevalent method for transmitting power to medical devices within WPT systems, encompassing near-field, mid-field, and far-field modes. These modes differ fundamentally in electromagnetic (EM) field characteristics and antenna dimensions. In the context of biomedical applications within the industrial, scientific, and medical (ISM) band, the near-field radio frequency (RF) signal experiences lower attenuation in human tissue [8], thus offering an efficient power transfer mechanism dependent on medical device size and power delivery requirements [9–13]. Near-field inductive WPT systems serve various purposes, including continuous powering of electronic medical devices [9–13] and temporary recharging of batteries [14,15] or super-capacitors [16,17]. These lightweight systems offer several benefits, such as eliminating the need for disposable batteries to reduce infection risks in implantable devices [18] and enabling continuous monitoring of vital signs like heart rate, respiratory rate, and body temperature without requiring human intervention, thereby allowing patients to maintain their normal daily activities and behaviors. A recent intriguing example showcased the enabling of skin-to-skin contact between parents and newborns, a vital aspect of the healing process for newborns, in neonatal intensive care unit (NICU) settings when wireless wearable sensors are deployed [19].

A WPT system fundamentally consists of a transmitter coil (Tx), a receiver coil (Rx), and a power amplifier (PA). The Tx coil produces an electromagnetic field to facilitate power transfer. The PA, integrated within the Tx, boosts the signal strength to ensure efficient power transmission. The Rx coil captures the electromagnetic field and converts it back into electrical power for the target device. Together, these components enable the effective wireless transfer of power.

Inductive links encounter challenges primarily due to the attenuation of mutual inductance, which occurs due to variations in coil distance or misalignment. This attenuation necessitates a high power transfer efficiency (PTE) to ensure adequate power delivery for intended applications. Various strategies have been proposed to address this challenge. For instance, the insertion of metamaterials (MTMs) between the Tx and Rx coils has been utilized to enhance efficiency [20]. MTMs are engineered materials with unique electromagnetic properties, such as wave amplification, which can potentially boost PTE [21]. The impact of MTMs on PTE improvement is discussed in [22]. Configuring MTMs, positioning them in the inductive link, and number of MTMs can enhance efficiency. In [23], two slabs of high-dielectric MTM resonators with cubic configuration are positioned in front of the Tx and Rx coils. This design leverages high-dielectric MTMs for superior efficiency, 58.5% improvement, and size reduction, 3.6 × 3.6 cm^2^, with high-dielectric materials. In [24], two slabs of spiral MTM resonators with square configuration are positioned at the two sides of Tx and Rx coils. It is demonstrated that side-positioned MTM slabs can significantly enhance WPT performance by 15.4%, achieving the same or better transmission efficiency as center-positioned slabs.

However, MTMs present several challenges compared to conventional inductive links [25]. For example, their intricate design complicates fabrication and integration, and they are sensitive to frequency shifts, which can affect performance stability. Moreover, the advanced manufacturing requirements lead to higher costs [26]. These issues can render MTMs challenging compared to simpler, cost-effective inductive coil systems [25]. Therefore, further research is needed to address these challenges associated with MTMs.

Moreover, multi-coil inductive links, discussed in [2,27–29], offer improved PTE performance over traditional two-coil setups, especially over longer distances. Recent advancements, including using multi-coil array structures [30,31], enable flexible coil positioning while improving efficiency.

Cage-based inductive link arrays have been introduced in experiments involving small animals [32–34]. These arrays are less sensitive to coil position and orientation, allowing animals to move freely within the cage with minimal impact on PTE. Enhanced PTE reduces heat dissipation and limits tissue exposure to strong magnetic fields, contributing to compliance with specific absorption rate (SAR) regulations [35,36]. Additionally, improved PTE minimizes power generation by the PA, which reduces interference with nearby communication devices [37]. In short, optimizing the efficiency of the entire WPT system is crucial to overcoming the mentioned challenges.

Given the significance of each component in the WPT system, careful consideration is necessary in PA design. Researchers explore various classes of PAs [38–40], providing comprehensive insights into design processes and equations. In inductive WPT systems, series configurations are common on the Tx side, particularly in biomedical WPT systems, as series configurations simplify impedance matching between the transmitter and receiver, enhancing PTE. Driving low impedance is essential in such configurations, making Class-E and -D PAs preferred options due to their high efficiency [3,41,42].

**Figure 1.**
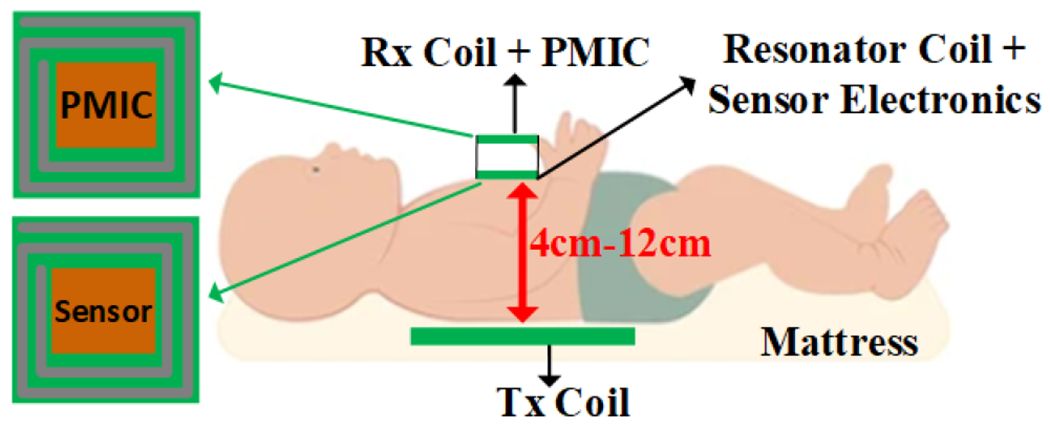
Conceptual set-up for a NICU application.

This study explores near-field inductive WPT systems, where resonance frequencies typically lie within the MHz range and operational distances span only a few centimeters [43,44]. A comprehensive review of previous advancements in biomedical WPT systems is provided and an in-depth analysis of the various parameters that influence the design of PAs and inductive links, accompanied by relevant equations. Additionally, a practical case study involving a three-coil inductive link operating at 13.56 MHz is presented, specifically tailored to the NICU applications, to illustrate a practical application of the theoretical principles governing each component. Figure 1 illustrates the conceptual three-coil WPT system for NICU set-up, where the Tx coil is embedded inside the mattress. The resonator and Rx coils are housed at a specific distance from each other according to the design requirement, and the distance from the Tx coil and resonator varies from 4 cm to 12 cm from patient to patient [45]. Rx coil and the power management integrated circuit (PMIC) are integrated on one board where PMIC regulates the power received by Rx based on the application power requirement. The regulated power is conducted to the wearable device through the via.

The remainder of this manuscript is organized as follows. Section 2 reviews the most commonly used PAs in biomedical WPT systems. Section 3 elaborates on the basic principles of induction in inductive links, while Section 4 discusses the important factors in coil design. Section 5 gives an overview of two-coil and multi-coil inductive links and compares them. Then, Section 6 presents a detailed case study on a three-coil inductive link, articulating the design details and sharing the results of experimental tests. Finally, Section 7 concludes the paper by summarizing the main findings.

## 2. Power Amplifiers for Wireless Power Transfer Systems

To enable the transmission of power through the inductive link in wireless power systems, a PA is employed within the transmitter. The PA serves as a DC-AC converter, addressing the inherent limitation of DC signals to propagate through the inductive link utilized in WPT systems. In contrast to their significance in audio applications, where considerations such as signal distortion and harmonics are dominating [46], the key specifications for PAs in WPT systems revolve around efficiency and delivered power [47].

PAs are classified into several classes based on their operational characteristics, with notable classes including class-A, -B, -AB, -C, -D, and -E. These classes are further categorized as either linear or switching amplifiers, contingent on their mode of operation. Linear amplifiers, encompassing Class-A, -B, -AB, and -C, function in accordance with the conduction angle of the input signal, thereby driving the active device, such as a bipolar junction transistor (BJT) or a metal-oxide-semiconductor field-effect transistor (MOSFET). Linear amplifiers are known for their inefficiency because the power transistor operates continuously throughout most, if not the entire, input signal cycle, leading to consistent current conduction. As a result, the amplifier consumes a substantial amount of power, even without an input signal, rendering it unsuitable for WPT applications [48–50].

Conversely, switching PAs, exemplified by Class-D and -E amplifiers, theoretically attain 100% efficiency under conditions of zero-voltage or zero-current switching [48]. Nonetheless, the efficiency of switching amplifiers is primarily compromised by losses and parasitic elements within the switching elements. Driven by square wave signals, these amplifiers experience efficiency degradation; however, such losses can be mitigated through the implementation of optimization techniques and adherence to specific operating conditions [42,51,52]. This section will articulate the operational and design principles of the most prevalent PA classes utilized in WPT systems, specifically Class-D and -E. Additionally, it will encompass an overview of cutting-edge implementations and optimization strategies geared toward maximizing efficiency.

### 2.1. Class-D Power Amplifier

**Figure 2.**
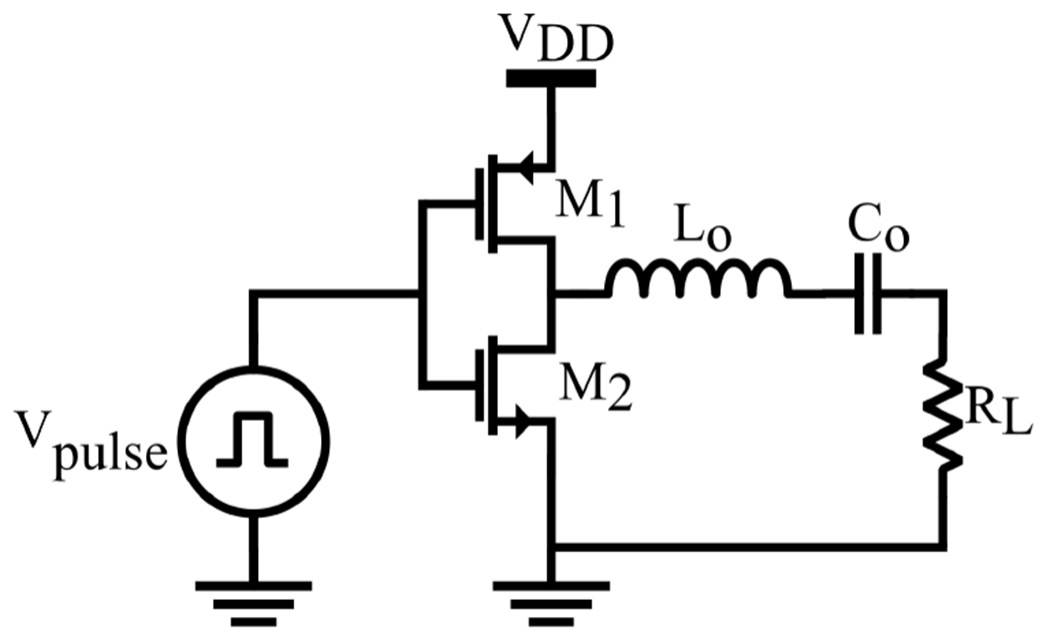
Simplified schematic of a Class-D power amplifier.

The Class-D PA, illustrated in Figure 2, first introduced in [53], consists of a pair of switching devices, *M*_1_ and *M*_2_, the resonant network, *L_o_* and *C_o_*, tuned at the desired carrier frequency, and a load resistor *R_L_*. The switching devices, commonly MOSFETs, act as a two-pole switch that operates alternatively between the supply and ground rails, generating a rectangular voltage or current waveform. The output circuit acts as a filter that removes the harmonics of the rectangular waveform, resulting in a sinusoidal output. While the ideal efficiency of a Class-D PA is 100%, losses in the switching devices make this theoretical value unattainable in practical implementations with losses reaching 30%. To achieve high efficiency, non-overlapping complementary signals must be used to drive the gates of the n-channel and p-channel MOSFET switches. This ensures the switches are never conducting simultaneously, which would cause significant losses in the PA. A comprehensive analytical model of the Class-D PA has been presented in [54,55].

**Figure 3.**
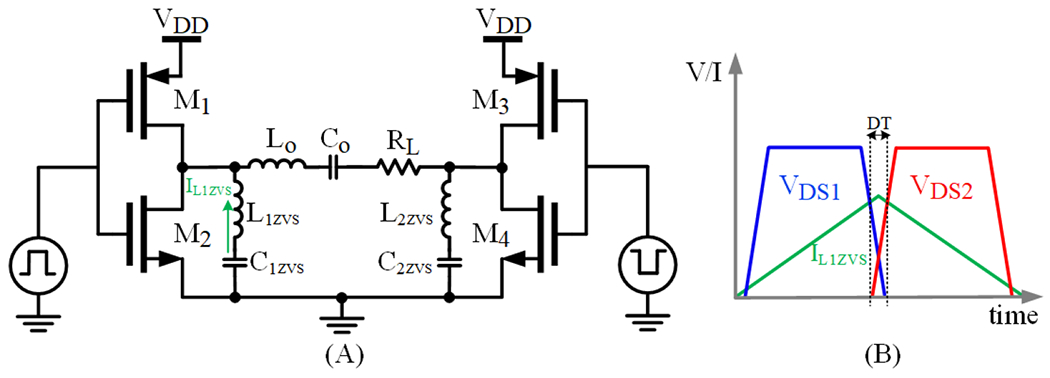
(**A**) Class-D full-bridge PA with ZVS LC tank, and (**B**) ideal waveforms.

In [56], a full-bridge Class-D PA with zero-voltage switching (ZVS) implementation has been proposed, illustrated in Figure 3A. The full-bridge amplifier topology uses two half-bridge inverters operating in a differential mode. ZVS is an operating mode that aims to improve PA efficiency by using a capacitor and an inductor, referred to as the ZVS tank, to smoothly transition the switching node voltage of the amplifier, which reduces the typical overlap losses due to switching device parasitic capacitances [57]. In the proposed PA, ZVS operating conditions are achieved while also implementing dynamic dead-time control (DDTC), which controls dead-time (DT) dynamically between the gate signals for each *V_DD_* value. The ideal waveforms for the ZVS tank’s current and the drain-source voltage of *M*_1_ and *M*_2_ switches are shown in Figure 3B. These conditions allow for reduced system losses by dynamically changing the DT to prevent large current spikes due to input voltage variation. This implementation can achieve higher steady-state efficiency and lower operating temperature than fixed DT PAs. Researchers in [58] present a similar PA with DT control along with the implementation of transistor width switching. Similar to [59,60], the transistor width switching scheme allows for improved efficiency over a wide load range by compromising between conduction losses and driving losses which are inversely proportional to each other based on transistor width. Using 1X, 2X, 4X, and 8X transistor sizes, small transistor widths are used for low-load currents, while larger widths are used for high-load currents.

In ZVS systems, the addition of the ZVS tank to achieve ZVS conditions results in the loading of the inductor appearing purely inductive. This causes the PA output current to lag behind the switching voltage, achieving ZVS operation. However, if the output current changes, the PA no longer operates at optimal ZVS conditions. While implementations such as [57] have attempted to improve this through an additional ZVS tank, the scheme relies on large inductor and capacitor values which makes it impractical for many applications. Ref. [61] proposes a discontinuous conduction mode (DCM) ZVS scheme for a Class-D PA. The proposed DCM PA uses additional switches in the ZVS tank and dual-loop control to adjust the turn-on time of the loops to achieve similar results as [57] while reducing the sizes of the ZVS inductor and capacitor.

PAs such as [62], for biomedical implants, are commonly required to provide constant voltage levels independent of the coil coupling. This can be achieved by implementing a control loop that adjusts the power delivered to the implant based on the coupling between the coils [63,64], or by adjusting the carrier frequency [65]. Table 1 compares WPT systems that have been implemented with a Class-D PA.

### 2.2. Class-E Power Amplifier

**Figure 4.**
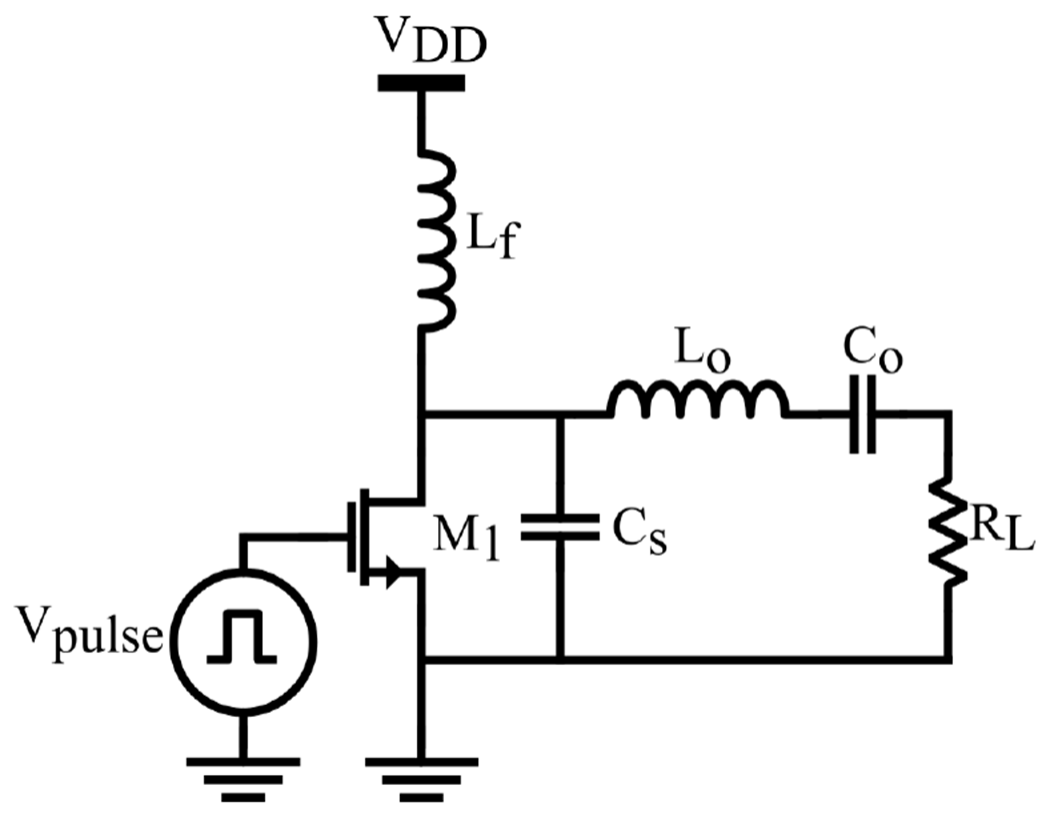
Simplified schematic of a Class-E power amplifier.

The Class-E PA was initially introduced in [66], highlighting the significance of minimizing the overlap of substantial current or voltage waveforms to mitigate switching losses within the PA. Due to its efficacy with just one switch element, the Class-E PA finds extensive application in biomedical and implantable devices. as illustrated in Figure 4, the fundamental circuit comprises a solitary n-channel power MOSFET, a shunt capacitor *C_s_*, a series resonance output circuit *L_o_* and *C_o_*, along with an RF choke inductor *L_f_*. *C_s_* ensures zero-voltage switching conditions for the non-ideal switch, while the output resonance network is tuned to the operational frequency, delivering a sinusoidal output. The gate of the MOSFET is driven by a square wave signal matching the operational frequency of the PA [67]. A comprehensive time domain analysis and explicit design equations for Class-E PA were presented in [68], showing that even with switch on-resistance, Class-E conditions remain achievable. Moreover, it suggests that substituting RF choke with finite DC-feed inductance can lead to efficiency improvements, with outcomes influenced by transistor technology and operational frequency.

Due to the reliance on resonant operation, the Class-E PA achieves maximum efficiency when the load value, *R_L_*, is carefully calculated. Deviating from the ideal value results in load-dependent output power, power efficiency, peak voltages, and peak currents, leading to reliability issues [69]. This is common in WPT systems, as the load resistance changes due to factors such as coil misalignment, which affects the coupling coefficient [70,71]. The most common solution to this problem is to implement a passive impedance matching network at the output of the PA. In [72], a modified Π matching network is implemented to increase the efficiency of the PA over a wide range of loads. This modification improves the amplifier’s efficiency from 50–90% to 80–88% over the load range of 1–100 Ω. Another solution is to replace the fixed passive circuit components with automatic tuning components, as discussed in [73,74]. Table 2 presents a comparison of WPT systems that have been implemented with a Class-E PA.

## 3. Principles of Induction

This section explores induction fundamentals critical for WPT systems, covering Faraday’s Law, self-inductance, mutual-inductance, and the coupling coefficient.

### 3.1. Faraday’s Law

**Figure 5.**
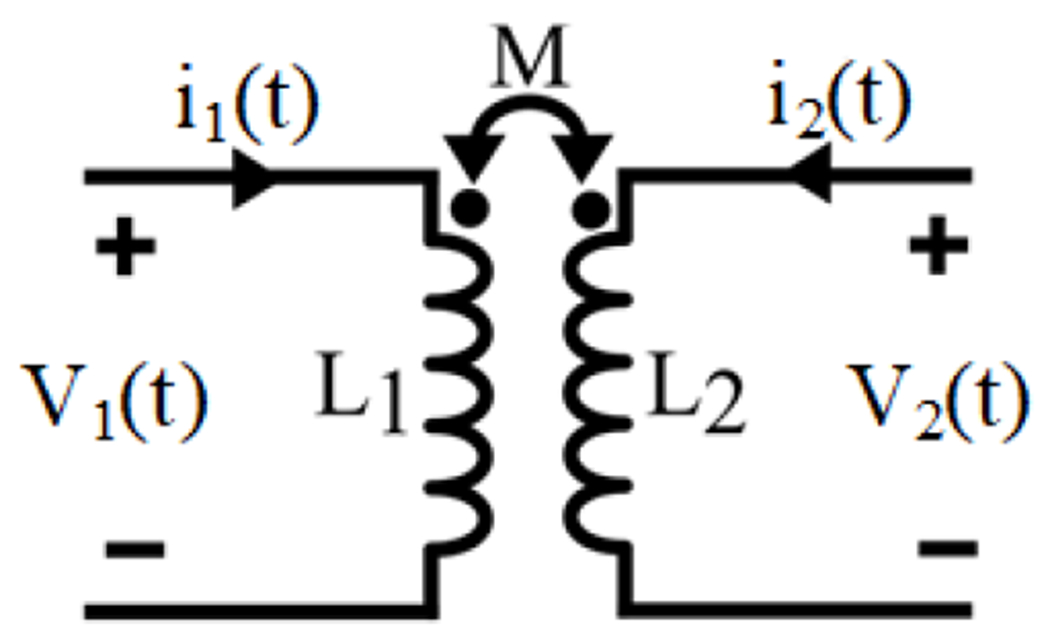
A generic architecture of an inductive WPT system.

The basics of WPT systems are built upon Faraday’s principle of electromagnetic induction. As illustrated in Figure 5, when a time-varying current, *i*_1_(*t*), flows through the primary coil *L*_1_, also known as Tx, it generates a time-varying magnetic field. The resulting magnetic field flux lines traverse the secondary coil *L*_2_, also known as Rx, inducing a voltage difference across Rx terminals. This voltage difference enables a time-varying current, *i*_2_(*t*), to flow through the *R_X_*.

### 3.2. Self-Inductance

When a time-varying current flows through a coil, it generates a magnetic field surrounding the conductor. This magnetic field creates an electromotive force (EMF) in the coil terminals that results in a voltage drop on the coil. v(t)=jωLi(t), where (ω=2πf), shows the relationship between the time-varying voltage and current with the self-inductance of a coil at the operating frequency of f. The inductance of a coil is measured in Henry (H), representing its ability to resist changes in the current.

### 3.3. Mutual-Inductance and Coupling Coefficient

The magnetic flux lines generated by the $T_{X}$ coil that passes through the $R_{X}$ coil can be quantified as the mutual inductance (M) representing the coupling strength between the coils. This can be translated as the part of the energy generated by the $T_{X}$ coil, which is received by the $R_{X}$ coil. Mutual inductance depends on the coils’ geometries, as well as the distance and alignment between the coils. A simplified closed-form expression for the mutual inductance for a pair of circular solenoid coils is

(1) $$M_{12}=\frac{\pi \mu_{0}n_{1}r_{1}^{2}n_{2}r_{2}^{2}}{2\sqrt{{\left(r_{1}^{2}+d^{2}\right)}^{3}}}\cos(\theta ),$$

where $\mu_{0}$ is the permeability of free space, $n_{1}$ and $n_{2}$ represent the number of turns, $r_{1}$ and $r_{2}$ are the radii of the coils, and d is the distance between the two coils. The angle θ represents the alignment between the center of two coils, where it becomes maximum when they are completely aligned. The mutual inductance is often normalized with respect to the coils forming the link as $k=\frac{M}{\sqrt{L_{1}L_{2}}}$, facilitating comparison and providing a clearer sense of the coupling between two coils. k can take values from 0 to 1, where a value close to 0 indicates a loosely coupled system, while a value close to 1 represents a strongly coupled system.

## 4. Coils

**Figure 6.**
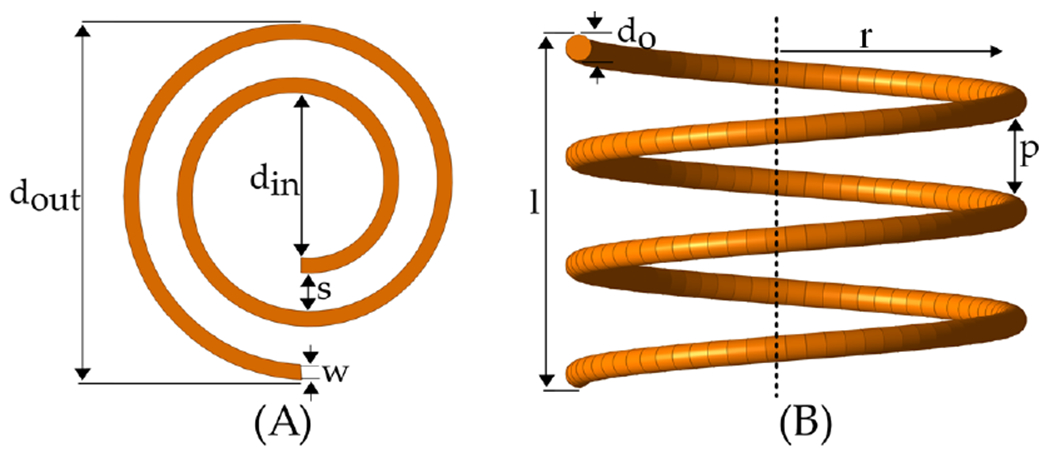
(**A**) Spiral and (**B**) helical coil structures with annotated parameters.

Based on the coils’ manufacturing method, they can be broadly categorized into two groups, printed spiral coils (PSC) and helical coils, as depicted in Figure 6. The selection of coil shape is primarily dictated by the specific application’s required geometry. For instance, PSCs offer easy customization and cost-effective mass production through lithography. They are preferred in situations where device thickness is limited, such as with credit cards, or when flexibility is needed to conform to curved surfaces like the human body. However, they face limitations such as difficulties in increasing the number of turns without expanding their diameter, leading to narrower conductive traces, higher equivalent series resistance (ESR), and lower Q-factor.

In contrast, helical coils cannot be mass-produced as economically and necessitate specialized winding equipment. Nonetheless, they generally face fewer restrictions and can offer significantly higher Q-factors as the cylindrical form of helical coils amplifies the concentration of magnetic fields. An advancement in the additive manufacturing electronic (AME) process [77] has introduced the capability to produce helical coils directly within substrates, enabling the creation of compact systems for miniaturized biomedical devices. It is important to note that this technology is not yet widely accessible; therefore, its cost and manufacturing time are not comparable to those of standard PSC manufacturing. The following sections will explain the essential equations for inductance, resistance, and parasitic capacitance of various geometries.

### 4.1. Self-Inductance

The self-inductance of a PSC coil depends on the geometric characteristics and is calculated in [78] using the following equations:

(2) $$\text{Circular:}\;L=\frac{\mu n^{2}d_{\text{avg}}}{2}\left(\text{ln}\left(\frac{2.46}{\phi }\right)+0.2\phi^{2}\right),$$

(3) $$\text{Square:}\;L=\frac{1.27\mu n^{2}d_{\text{avg}}}{2}\left(\text{ln}\left(\frac{2.07}{\phi }\right)+0.18\phi +0.13\phi^{2}\right),$$

(4) $$\text{Hexagonal:}\;L=\frac{1.09\mu n^{2}d_{\text{avg}}}{2}\left(\text{ln}\left(\frac{2.23}{\phi }\right)+0.17\phi^{2}\right),$$

(5) $$\text{Octagonal:}\;L=\frac{1.07\mu n^{2}d_{\text{avg}}}{2}\left(\text{ln}\left(\frac{2.29}{\phi }\right)+0.19\phi^{2}\right),$$

where $\mu =\mu_{r}\cdot \mu_{o}$ represents the permeability that for nonmagnetic materials, $\mu_{r}$ is assumed to be 1. The average diameter is expressed as $d_{\text{avg}}=\frac{d_{\text{out}}+d_{\text{in}}}{2}$, and n is the number of turns. The fill factor (ϕ), ranging from 0 to 1, depends on the amount of area filled with the conductor that is calculated through $\phi =\frac{d_{\text{out}}-d_{\text{in}}}{d_{\text{out}}+d_{\text{in}}}$.

The self-inductance of a helical coil is obtained through the following equation [79]:

(6) $$L=\frac{\mu_{0}\pi r^{2}n^{2}\kappa }{l},$$

where $\mu_{0}$ is the permeability of the free space, r is the coil radius, n is the number of turns, l is the coil length, and κ is the Nagaoka’s coefficient obtained through the following equation:

(7) $$\kappa =z_{k}\left(\text{ln}\left(1+\frac{1}{z_{k}}\right)+\frac{1}{\left(k_{0}+k_{1}\left(\frac{l}{d}\right)+k_{2}{\left(\frac{l}{d}\right)}^{2}+\frac{w_{1}}{{\left({\left|w_{2}\right|}^{2}+d/l\right)}^{v}}\right)}\right),$$

where $\left.z_{k}=l/(\pi r)\right),k_{0},k_{1},k_{2}$ refer to the correction coefficients and have the following values; $k_{0}=2.30038,k_{1}=3.437,k_{2}=1.76356,w_{1}$ and $w_{2}$ are Weaver’s coefficients to reduce the error and they have the following values; $w_{1}=-0.47,w_{2}=0.755$, and v=1.44 [3,79].

### 4.2. Parasitic Resistance

The parasitic resistance of a coil is divided into DC resistance and AC resistance. $R_{DC}$, is calculated straightforwardly through $R_{DC}=\frac{\rho l}{A}$ as it primarily depends on material resistivity (ρ), the conductor cross-section area (A), and the length of the coil (l).

In contrast to its DC counterpart, the AC resistance is influenced by an additional frequency factor, contributing to its computational intricacy. Within the realm of PSCs, two significant phenomena associated with AC resistance are the skin effect and the proximity effect. The skin effect, symbolized as $R_{\mathrm{skin}}$ and formulated in Equation (8), exhibits a strong reliance on parameters such as frequency (ω), trace width (w), and thickness $\left(t_{0}\right)$. With increasing frequency, a skin depth (δ) is established according to Equation (9), and begins to form around the conductor that represents the effective cross-sectional area through which the current can flow.

(8) $$R_{skin}=R_{DC}\frac{t_{0}}{\delta \left(1-e^{-\frac{t_{0}}{\delta }}\right)}\frac{1}{1+\frac{t_{0}}{w}}.$$

(9) $$\delta =\sqrt{\frac{2\rho }{\omega \mu }}.$$

The proximity effect exacerbates the coil resistance, denoted as $R_{\mathrm{prox}}$, arising from the magnetic field generated by the interaction of current flow in neighboring traces within the coil. This magnetic field induces eddy currents, which in turn elevate the coil resistance. The approximate estimation of $R_{\mathrm{prox}}$ is given as follows

(10) $$R_{\text{prox}}=\frac{R_{DC}}{10}{\left(\frac{\omega }{\omega_{\text{crit}}}\right)}^{2},$$

where $\omega_{\mathrm{crit}}$ is the critical frequency and signifies the frequency threshold at which current crowding prevails and the proximity effect becomes dominant [80]. It is calculated as

(11) $$\omega_{\text{crit}}=\frac{3.1}{\mu_{0}}\frac{(w+s)\rho }{w^{2}t_{0}},$$

where *s* is the spacing between the adjacent traces.

Ultimately, the total ESR, *R_s_*, is derived by combining the resistances attributed to both the skin effect and the proximity effect.

(12) $$R_{s}=R_{\mathrm{skin}}+R_{\mathrm{prox}}.$$

### 4.3. Parasitic Capacitance

The gap between traces inadvertently forms a capacitor in parallel with the coil, reducing its self-resonant frequency. It is important to evaluate this frequency to ensure it remains well separated from the operating frequency. Otherwise, the stability of the inductance value could be compromised, presenting difficulties in circuit tuning. Two types of insulating materials influence the value of parasitic capacitance. The first type is the material between two adjacent traces, which can be air or another coating. The parasitic capacitance due to this material is denoted as $C_{pc}$. The second type is the substrate, which can be ceramic, FR4, or polyimide. The parasitic capacitance associated with the substrate is denoted as $C_{ps}$. Ref. [81] has proposed an equation to estimate the parasitic capacitance, which includes these two parameters:

(13) $$C_{P}=C_{pc}+C_{ps}\approx \left(\alpha \varepsilon_{rc}+\beta \varepsilon_{rs}\right)\varepsilon_{0}\frac{t_{0}}{s}l_{g},$$

where $\varepsilon_{0}$ is the vacuum permittivity, $l_{g}$ is the length of the gap, which can be estimated through Equation (14). $\varepsilon_{rc}$ and $\varepsilon_{rs}$ represent the relative permittivity of the coating and substrate materials, respectively. Assuming the coating is air and the substrate is FR4, the coefficients α and β for these components are 0.9 and 1.1, respectively. Furthermore, the values of $\varepsilon_{rc}$ and $\varepsilon_{rs}$, are 1 and 4.4, respectively.

(14) $$l_{g}=4\left(d_{out}-w\cdot n\right)(n-1)-4s\cdot n(n+1).$$

### 4.4. Quality Factor (Q)

The quality factor of a coil is determined by comparing its reactance, which includes parasitic elements contributing to the overall reactance, to its parasitic resistance. To achieve a strong magnetic field with a specific inductance, a high value of *Q* is necessary. A *Q* value less than 1 indicates that losses dominate over the inductive properties. For a well-designed coil for a particular frequency, *Q* peaks at the desired frequency and then drops close to the self-resonance frequency [82]. The quality factor, estimated in [81], is

(15) $$Q=\frac{\omega L-\omega \left(R_{S}^{2}+\omega^{2}L^{2}\right)C_{P}}{R_{S}}.$$

### 4.5. Mutual Inductance for PSC

To determine the mutual inductance between two PSC coils, the turns of each coil are conceptualized as a series of concentric loops. The individual mutual inductance between the *i^th^* turn of one coil and *j^th^* turn of the other coil is calculated as

(16) $$M_{ij}=\frac{2\mu }{\alpha }\sqrt{r_{i}\cdot r_{j}}\left[\left(1-\frac{\alpha^{2}}{2}\right)K(\alpha )-E(\alpha )\right],$$

where $r_{i}$ and $r_{j}$ denote the radius of each turn, K(α) and E(α) represent the complete elliptic integral of the first and second kind, respectively, and α is derived as

(17) $$\alpha =2\sqrt{\frac{r_{i}\cdot r_{j}}{{\left(r_{i}+r_{j}\right)}^{2}+D^{2}}},$$

where *D* is the center-to-center distance between the coils. The total mutual inductance is obtained by adding up the individual mutual inductance between each turn of the coils as

(18) $$M=g\sum_{i=1}^{n_{1}}\sum_{j=1}^{n_{2}}M_{ij}\left(r_{i},r_{j},D\right),$$

where the parameter *g* is the correction factor determined based on the coil geometry and estimated in [81]. For a pair of hexagonal coils, *g* is 0.95; for a pair of circular coils, it is 1.0; and for a pair of square coils, it is 1.1.

## 5. Inductive Link

The inductive link in a WPT system serves as a fundamental component for transmitting power wirelessly over a distance. This section investigates two primary configurations: two-coil and multi-coil inductive links. It explores the core principles, efficiency, and power equations, as well as the inherent advantages and drawbacks of each configuration. A robust understanding of these technical aspects is necessary for the optimal design and performance enhancement of inductive links across various WPT applications.

### 5.1. Two-Coil Inductive Link

**Figure 7.**
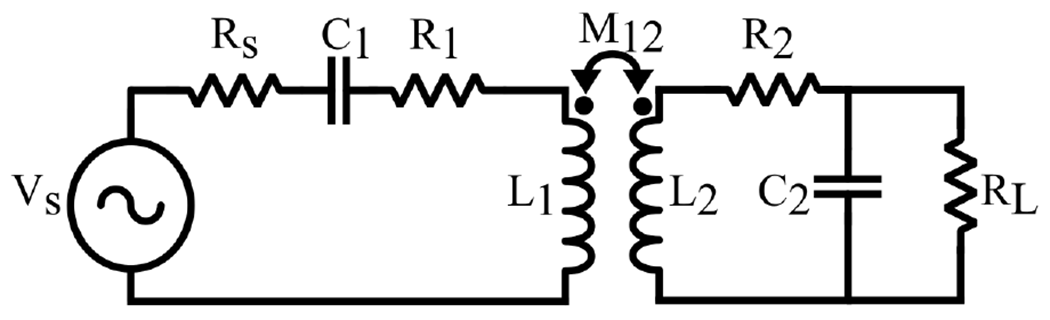
A basic structure of a two-coil resonance inductive link.

A two-coil inductive link topology, as depicted in Figure 7, is optimal for the short coupling distances between the Tx and Rx coils and is suitable when a high power delivered to load (PDL) is necessary [2,9,83]. This configuration includes two inductors and two resonance capacitors, all tuned to the same resonance frequency as the carrier signal. The coupling coefficient influences the impedance on both the Tx and Rx sides. To understand its effect on the Tx side impedance, we must consider the reflected impedance, $R_{ref}$, from the Rx to the Tx. The power delivered to the $R_{ref}$ indicates the power received by the Rx side. Thus, the energy transfer from the Tx to the Rx occurs through the $R_{ref}$ [44,84]. $R_{ref}$ is calculated as

(19) $$R_{ref}=k_{12}^{2}R_{1}Q_{1}Q_{2L},$$

where $R_{1}$ is the series loss resistance of the Tx coil, $k_{12}$ is the coupling coefficient, $Q_{1}$ is the quality factor of the Tx coil, $Q_{2L}=\frac{Q_{2}Q_{2L}}{Q_{2}+Q_{L}}$ is the loaded quality factor of Rx coil, where $Q_{2}=\frac{\omega_{0}L_{2}}{R_{2}}$ is the quality factor of Rx coil, $R_{2}$ is the series loss resistance of the Rx coil, and $Q_{L}=\frac{\omega_{0}L_{2}}{R_{L}}$ is the quality factor of the load. The efficiency can be divided into two parts—the Tx efficiency and the Rx efficiency, which are calculated through the following equations:

(20) $$\eta_{Tx}=\frac{k_{12}^{2}Q_{1}Q_{2L}}{1+k_{12}^{2}Q_{1}Q_{2L}},$$

(21) $$\eta_{Rx}=\frac{Q_{2}}{Q_{2}+Q_{L}}.$$

The overall efficiency of the two-coil inductive link can be obtained as

(22) $${\mathrm{PTE}}_{\left(2–coil\right)}=\eta_{Tx}\cdot \eta_{Rx}=\frac{k_{12}^{2}Q_{1}Q_{2L}}{1+k_{12}^{2}Q_{1}Q_{2L}}\frac{Q_{2}}{Q_{2}+Q_{L}}.$$

The PDL can be calculated by multiplying the PTE by the power generated by *V_s_* signal source as

(23) $$PDL_{\left(2–\text{coil}\right)}=PTE_{\left(2–\mathrm{coil}\right)}\cdot \frac{Vs^{2}}{2\left(R_{ref}+R_{1}\right)}=\frac{V_{s}^{2}}{2R_{1}}\cdot \frac{k_{12}^{2}Q_{1}Q_{2L}}{{\left(1+k_{12}^{2}Q_{1}Q_{2L}\right)}^{2}}.$$

**Figure 8.**
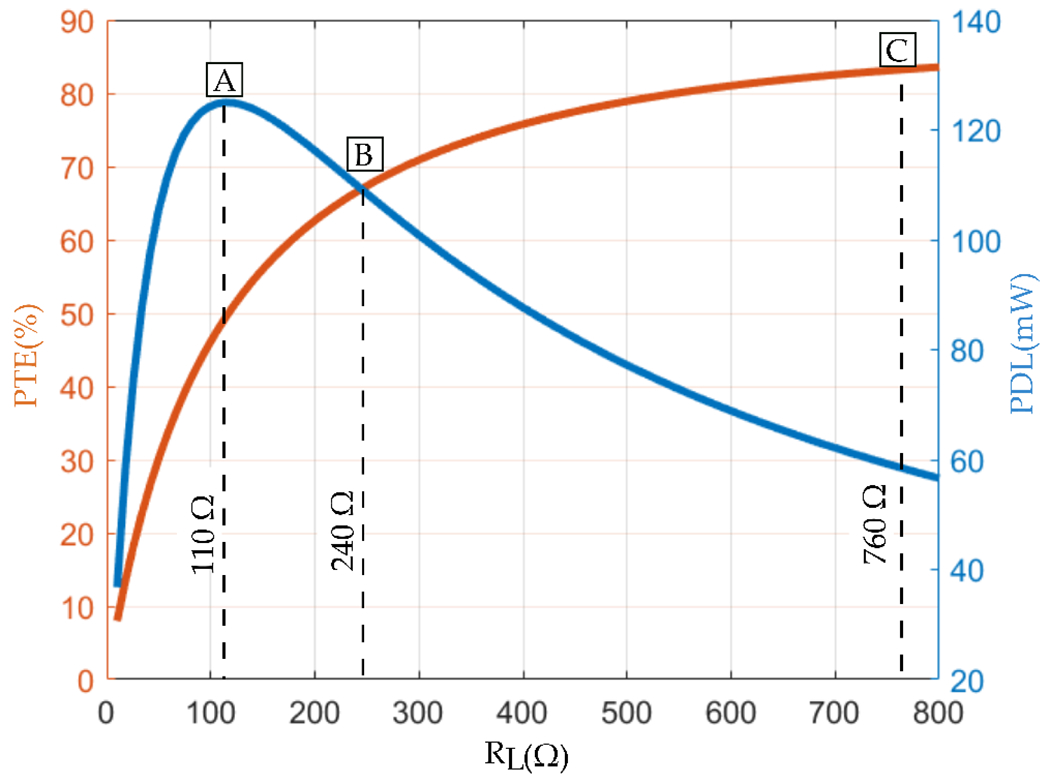
Load condition for a two-coil inductive link and its influence on both PTE and PDL.

We conducted a calculation in MATLAB to illustrate that a two-coil inductive link cannot maximize PTE and PDL simultaneously. The analysis shows that while a load of 760 Ω maximizes PTE (marker C), a load of 110 Ω maximizes PDL (marker A), as shown in Figure 8. This discrepancy indicates the design’s failure to optimize both parameters at the same load. Adjustments in coil geometries and loading conditions suggest a compromise load of 240 Ω (marker B). However, this highlights the challenge of balancing PTE and PDL, emphasizing the incapability of the two-coil inductive link design for simultaneous optimization. For this purpose, multi-coil inductive links will be studied in the following section.

### 5.2. Multi-Coil Inductive Link

Multi-coil inductive links, characterized by having more than two coils, enable maintaining high PTE over extended distances between Tx and Rx coils [2,85,86]. Enhanced PTE in such instances correlates with reduced heat dissipation within tissues [29,87,88]. This attribute proves particularly valuable in scenarios where SAR poses constraints. For prolonged experiments, such as those involving EnerCages with rodents, where balancing high PTE with a reasonable PDL is crucial, four-coil inductive links are commonly favored [32,34,86,89].

Our analysis will focus on the three-coil inductive link, which serves as the cornerstone for developing our case study in the subsequent section. The motives and technical reasons behind this decision can be explained by the fact that the three-coil inductive link has superiority over the two-coil inductive link in terms of PTE and PDL at large power transfer distances [2]. Additionally, the three-coil inductive links can significantly improve the PTE and PDL, particularly at large coupling distances, by transforming any arbitrary load impedance to the optimal impedance at the Tx side. This is due to the coupling between *L*_2_ and *L*_3_, which provides the designer with flexibility for impedance transformation. This coupling acts as a matching network, altering the reflected impedance on the resonator and, consequently, on the Tx side, resulting in improved efficiency. However, a four-coil inductive link transforms the load impedance into a large reflected impedance at the Tx side, which can load the output of the signal generator, leading to degraded PDL compared to the three-coil inductive link. Moreover, the three-coil inductive link uses fewer components than the four-coil configuration, which reduces the complexity of the design.

**Figure 9.**
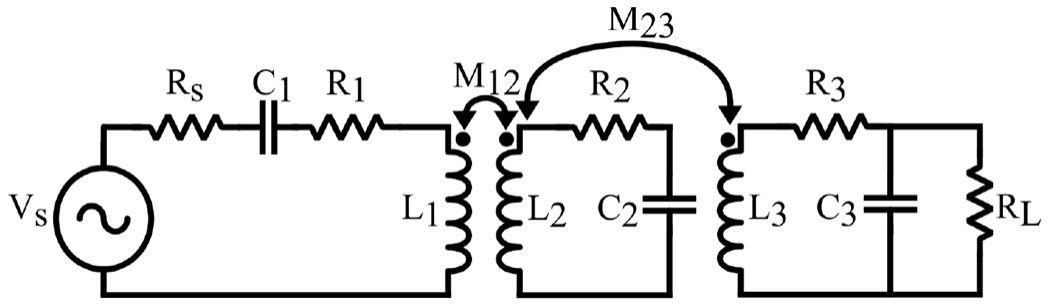
A basic structure of a three-coil resonance inductive link

This link consists of a Tx coil $\left(L_{1}\right)$, a resonator coil $\left(L_{2}\right)$, and a Rx coil $\left(L_{3}\right)$ as illustrated in Figure 9. The separation between $L_{1}$ and $L_{2}$ is greater than that between $L_{2}$ and $L_{3}$, leading to a small mutual inductance between $L_{1}$ and $L_{2},M_{12}$. The coupling between $L_{1}$ and $L_{3}$ is negligible and is disregarded to simplify the equations, given its minimal effect. Coils $L_{2}$ and $L_{3}$ are positioned close together and are referred to as the Rx stage. The efficiency equations of the three-coil inductive link are represented by Equation (24) [2], where the first term denotes the Tx efficiency, the second term denotes the resonator efficiency, and the third term denotes the Rx efficiency. PDL is expressed by Equation (25) [2], which is derived by multiplying the three-coil inductive link PTE by the power generated by the signal source.

(24) $$PTE_{\left(3\text{-coil}\right)}=\frac{k_{12}^{2}Q_{2}Q_{1}}{k_{23}^{2}Q_{2}Q_{3L}+k_{12}^{2}Q_{1}Q_{2}+1}\frac{k_{23}^{2}Q_{2}Q_{3L}}{k_{23}^{2}Q_{2}Q_{3L}+1}\frac{Q_{3L}}{QL}.$$

(25) $$PDL_{\left(3\text{-coil}\right)}=\frac{V_{s}^{2}}{2R_{1}}\frac{Q_{3L}}{Q_{L}}\frac{\left(k_{23}^{2}Q_{2}Q_{3L}\right)\left(k_{12}^{2}Q_{1}Q_{2}\right)}{{\left(k_{23}^{2}Q_{2}Q_{3L}+k_{12}^{2}Q_{1}Q_{2}+1\right)}^{2}}.$$

In these equations, $Q_{3}$ is the quality factor of the Rx coil, $Q_{3L}$ is the loaded quality factor of the Rx coil, $k_{12}$ is the coupling coefficient between the Tx and resonator coils, and $k_{23}$ is the coupling coefficient between the resonator and Rx coils.

**Figure 10.**
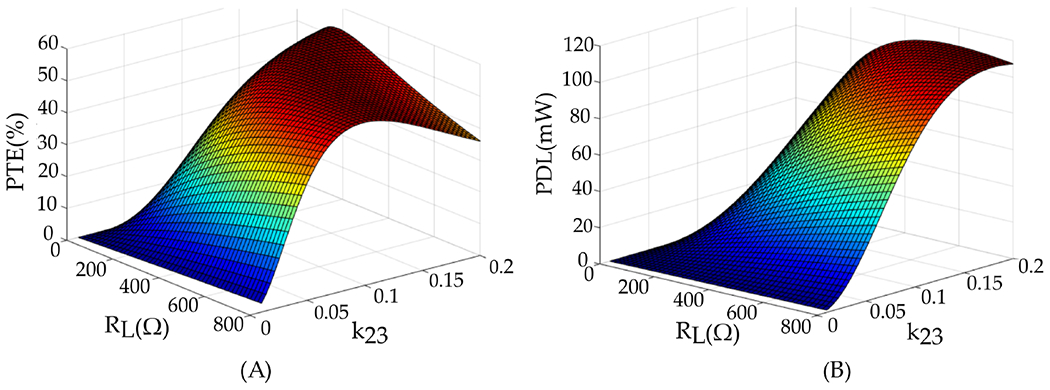
Analysis of (**A**) PTE and (**B**) PDL in a three-coil inductive link involves adjusting *k*_23_ and *R_L_*.

Figure 10 was generated utilizing Equations (24) and (25) in MATLAB. For a specific set of coils with known geometric parameters chosen to fulfill the NICU application requirements, the coupling coefficients are calculated through Equations (16)–(18). The coupling coefficient *k*_12_ is calculated and set to 0.02 when the distance is around 10 cm between the Tx and resonator coils. These plots illustrate that by adjusting the distance between the resonator and Rx coils, achieving high PTE and PDL simultaneously is possible when the coupling coefficient *k*_23_ is within the range of 0.12 to 0.18. Additionally, these conceptual plots highlight the applicability of the three-coil inductive link configuration in long-distance scenarios. As discussed previously, the primary advantage of this inductive link configuration lies in the effective manipulation of the *k*_23_ coupling.

## 6. A Three-Coil Inductive Link Case Study

This case study aims to design a WPT system for powering blood gas monitoring sensors in NICU applications. The system is intended to avoid wired systems that hinder skin-to-skin contact between parents and infants and eliminate the need for batteries, allowing continuous monitoring. The sensor is designed to be placed on the newborn’s chest. Hospital data indicate that the anteroposterior (AP) diameter of newborns ranges from 4 cm to 12 cm; similarly, if the sensor is placed on the calf, the diameter also ranges from 4 cm to 12 cm [45]. This section will detail the design procedure of a three-coil inductive link to provide sufficient power at a maximum distance with high PTE by taking into consideration SAR regulations. Additionally, measurement techniques for key parameters of the inductive link will be explained, and experimental results will be presented. It is useful to mention that the maximum memory usage in HFSS for the optimization process was 6.68 GB. Memory usage varies from task to task in HFSS. For instance, initial meshing requires 46 MB memory, while matrix solving uses the maximum memory.

### 6.1. Inductive Link Design

Taking into account the operational distance and the area constraints imposed by the encapsulation of the blood gas monitoring sensor, achieving adequate PDL and high PTE presents a significant design challenge. The three-coil configuration is the most suitable for NICU application, visualized in Figure 1, as its technical reasons were explained in the previous section. Since the Tx coil will be placed in a mattress, this placement provides more flexibility for the Tx coil design in terms of area and structure. For this configuration, we employ a high-Q Tx coil as proposed in [2], while the Rx and resonator coils are designed to meet the application’s specifications, ensuring the limited area and required power transfer to the Rx coil even at the maximum distance from the Tx coil. The outer diameter of the resonator coil, $L_{2}$, is 5 cm, and the Rx coil, $L_{3}$, is up to 2.5 cm. To initiate the design iteration, we assume values for the coil geometries to calculate the coupling coefficients using a MATLAB code based on the coupling coefficient equations. This process yields an initial PTE value, and subsequent adjustments to the coil geometries are made to achieve optimal efficiency. Based on the power requirements and average current drawn by the sensor, a 500 Ω load resistor represents the sensor in our study.

**Figure 11.**
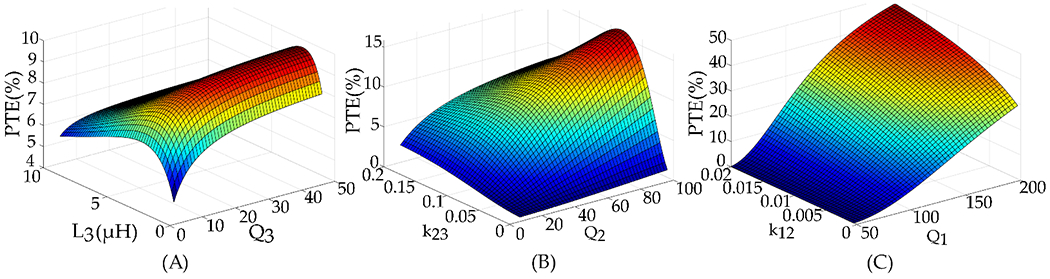
Effect of (**A**) *L*_3_ and Q_3_, (**B**) *k*_23_ and *Q*_2_, and (**C**) *K*_12_ and *Q*_1_ on PTE of the 3-coil inductive link.

To conduct this analysis, simulations were performed in HFSS to estimate the quality factors for resonator and Rx coils, and MATLAB was used to calculate the efficiency of the inductive link based on the resulting quality factors. Figure 11 illustrates our approach to the design and the modification of the inductive link. Initially, we investigated the influence of the Rx coil on PTE. Figure 11A demonstrates how the efficiency of the link depends on the inductance and the quality factor values of the Rx coil, $L_{3}$ and $Q_{3}$, respectively. Both values should be maximized to achieve better efficiency, with inductance being more dominant compared to the quality factor. Although the efficiency peak is around 4 μH, the maximum inductance value for the designed Rx coil is 3.06 μH due to area constraints.

To study the effect of the resonator coil on the efficiency of the link, we analyzed the impact of the resonator coil, plotting PTE versus $Q_{2}$ and $k_{23}$ in Figure 11B. According to the plot, the optimal PTE occurs when $k_{23}$ is equal to 0.09, and $Q_{2}$ is maximized as much as possible. The final step in designing the resonator coil is studying the effect of the Tx coil and the coupling coefficient between the Tx coil and resonator on the efficiency. As demonstrated in Figure 11C, it is crucial to maximize both $Q_{1}$ and $k_{12}$. Therefore, the resonator coil should be designed to achieve the highest possible $Q_{2}$ and $k_{12}$. The optimal value for $k_{23}$ can be adjusted later by changing the separation distance between $L_{2}$ and $L_{3}$.

**Figure 12.**
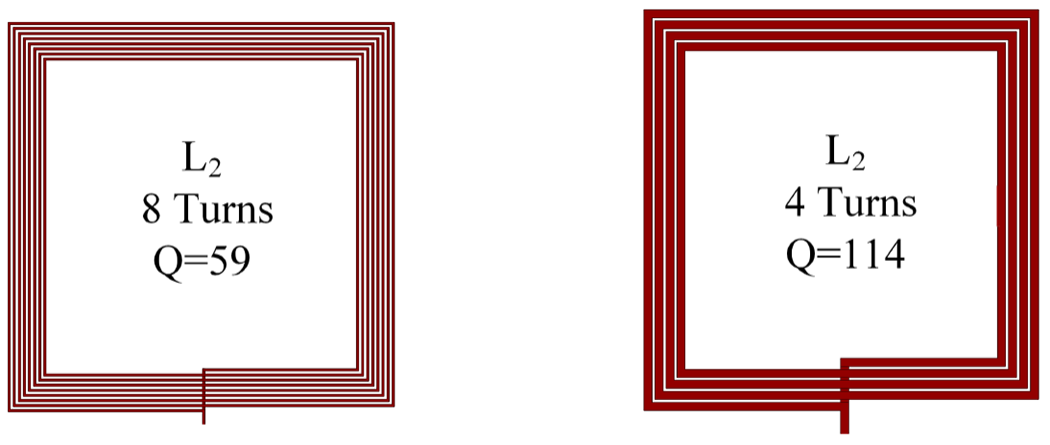
Two different designs for resonator coils.

**Figure 13.**
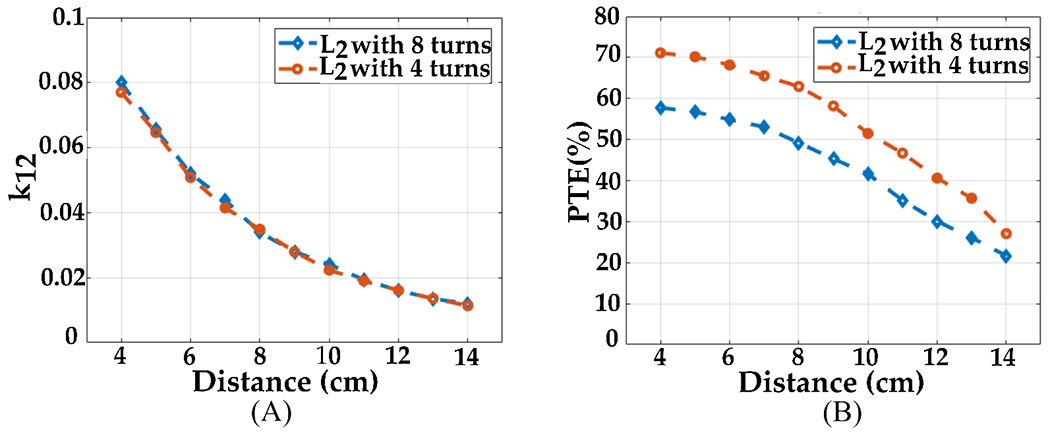
Simulation results showing the effect of 2 different designs for the resonator coils *L*_2_ on the (**A**) coupling coefficient (*k*_12_), (**B**) and efficiency (PTE).

For the preliminary design of $L_{2}$, the initial design had 8 turns, while the final design has four turns and wider traces as shown in Figure 12. Figure 13A demonstrates that both configurations have a similar coupling coefficient since the transmitter size dominates compared to the resonator when the distance between the Tx and resonator ranges from 4 cm to 14 cm. However, in Figure 13B, the PTE of the inductive link improves with a higher quality factor of the resonator. Additionally, we designed the transmitter Tx with fewer turns and wide traces to achieve a high-quality factor. As a result, the Tx $L_{1}$ demonstrated an impressive quality factor of 173 and an inductance value of 1.1 μH.

### 6.2. Quality Factor Measurement

**Figure 14.**
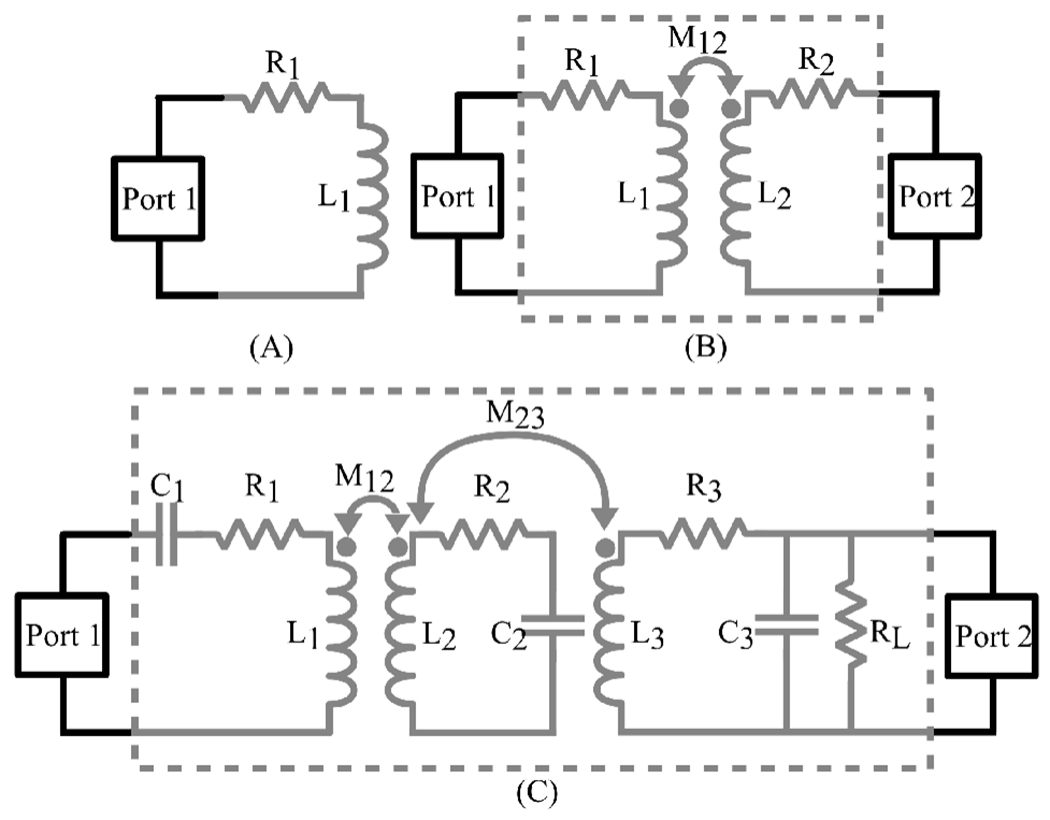
Measurement configuration for, (**A**) quality factor, (**B**) coupling coefficient, (**C**) PTE.

The geometries and specifications of the fabricated coils are detailed in Table 3. The quality factors of the coils were measured using a KEYSIGHT vector network analyzer (VNA) at 13.56 MHz. The quality factors were determined through a one-port measurement, where one port of the network analyzer was directly connected to the coil to measure the $S_{11}$ parameter as shown in Figure 14A. This parameter was then converted to $Z_{11}$ and embedded in the following equation:

(26) $$Q=\frac{Im\left(Z_{11}\right)}{Re\left(Z_{11}\right)}.$$

### 6.3. Coupling Coefficient Measurement

For measuring the coupling coefficient between Tx and resonator, one port was connected to $L_{1}$, and the other to $L_{2}$ as shown in Figure 14B. We also used the same method to measure the coupling coefficient between the resonator and Rx. The S-parameters for each distance point were extracted using the network analyzer and converted to Z-parameters. We employed the following equation to obtain the coupling coefficient value:

(27) $$k=\sqrt{\frac{Im\left(Z_{12}\right)Im\left(Z_{21}\right)}{Im\left(Z_{11}\right)Im\left(Z_{22}\right)}}.$$

**Figure 15.**
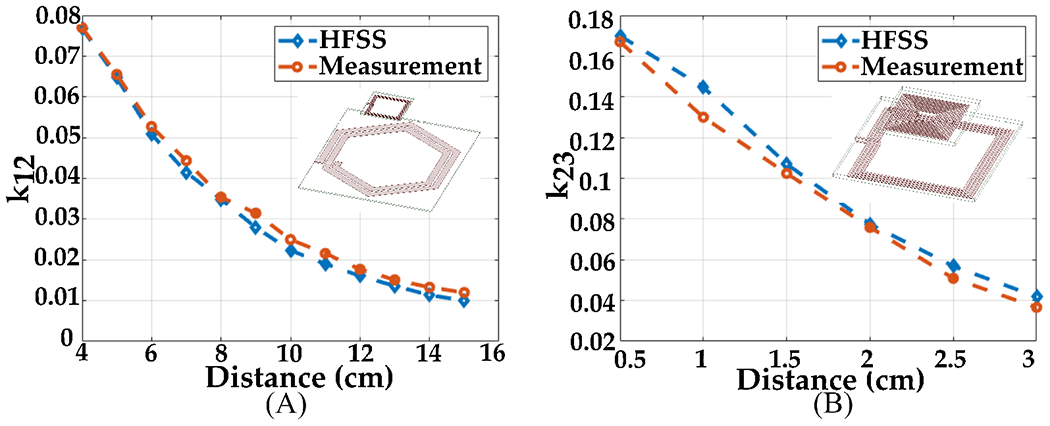
Measurement and simulation results for the coupling coefficient between (**A**) Tx and resonator, and (**B**) Rx and resonator.

Figure 15 illustrates the measurement and the simulation results for the coupling coefficient between the designed coils. We observed a close agreement between measurement results and HFSS simulation results.

We acknowledge that the coupling coefficient is a crucial parameter for achieving high PTE and PDL. One potential method to enhance the coupling coefficient for future iterations is to increase the size of the coils. However, due to the spatial constraints of the NICU application, the size of the receiver stage coil is limited. As a result, the Tx coil can be designed to be as large as the mattress’ size, ensuring that the magnetic field covers the entire area and provides robust coupling. Another approach is to develop a design where the Tx coil is integrated into the walls of the incubator, wrapping around the incubator’s sidewalls and incorporating the Tx coil within the mattress. This configuration could potentially improve coupling across the entire incubator space.

### 6.4. PTE Measurement

**Figure 16.**
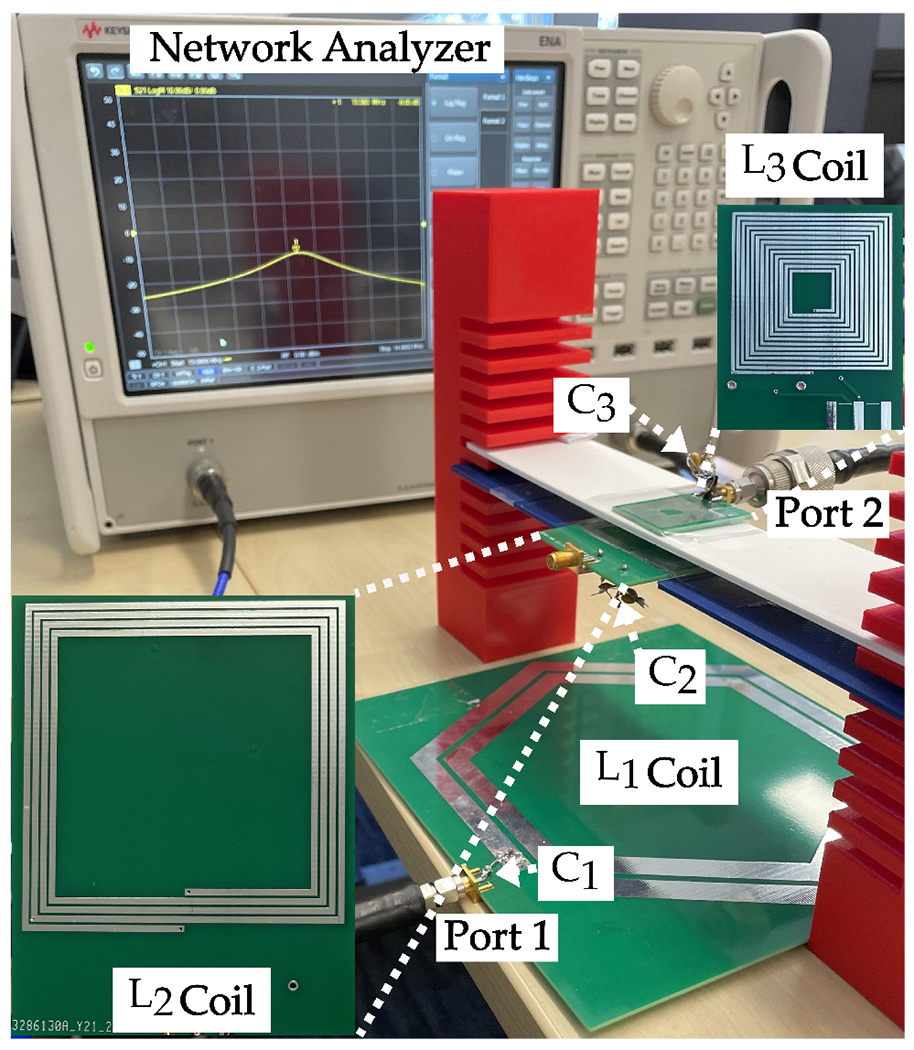
PTE measurement setup.

To conduct the PTE measurement, we followed the setup detailed in [2]. Initially, the inductive link was tuned to 13.56 MHz by connecting the resonance capacitor to each coil. As the resonator and Rx coils are positioned very close to each other, the increased mutual inductance between these coils becomes comparable to the inductance values, leading to a resonance frequency shift that can affect the PTE of the WPT system [43]. To mitigate this effect, it is essential to tune all coils together at the resonance frequency when coupled, as tuning each LC tank individually is ineffective. Variable capacitors were used to determine the correct resonance capacitor values for each coil by observing the system’s resonance frequency using a VNA. Subsequently, fixed capacitors were implemented in the system. The resonance capacitors used in the inductive link deviated by ∼±20% from the values calculated using the resonance equation $f=\frac{1}{2\pi \sqrt{LC}}$. After tuning the system to the resonance frequency, the network analyzer’s ports were connected to the inductive link according to Figure 14C. The PTE measurement setup is demonstrated in Figure 16. The S-parameters for each distance point were obtained from the network analyzer and converted to Z-parameters. Finally, we employed the following equation to calculate the PTE for each distance point:

(28) $$\mathrm{PTE}=\frac{{\left|Z_{21}\right|}^{2}}{\left|Z_{11}\right|\cos\left(∠Z_{11}\right)}.$$

**Figure 17.**
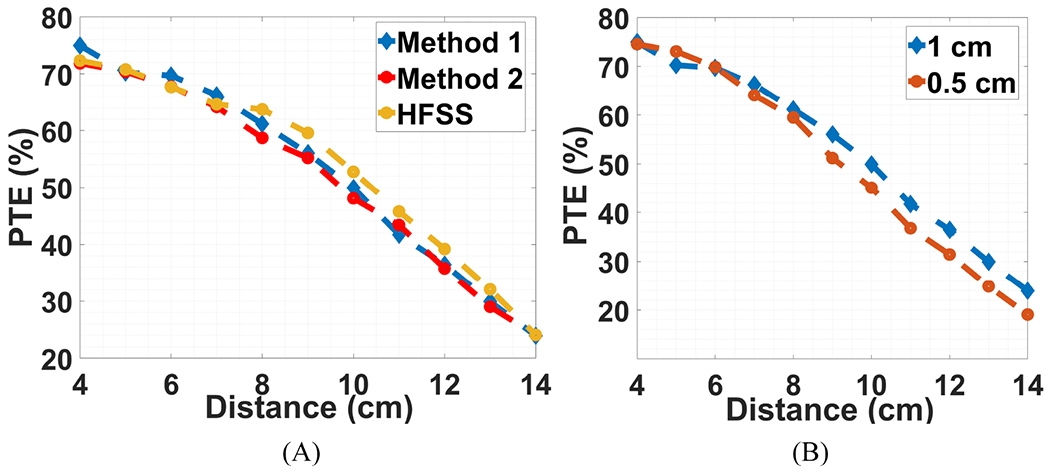
(**A**) Comparison of the results obtained with different methods, (**B**) comparison of different distances between the resonator and Rx.

The results obtained with this calculation method are shown in Figure 17A, labeled as “Method 1”. To verify these results, we also used Equation (24) by inputting the measured quality factor of each coil and their respective coupling coefficients for each distance point. The results obtained with this method are depicted in Figure 17A, labeled as “Method 2”. Additionally, the results obtained from HFSS simulations are presented in the same figure.

At the beginning of the measurement stage, we encountered a discrepancy between Method 1 and Method 2. Upon closer examination of the input impedance of the transmitter resonance tank, we identified that the series resonance ceramic capacitor was introducing approximately 3.5 Ω of resistance. This additional resistance led to a degradation in the magnitude of Z_11_ and its phase angle, resulting in a decline in PTE. Subsequently, by substituting the ceramic capacitor with a surface mount device (SMD) capacitor, characterized by lower parasitic resistance, we achieved the anticipated input impedance on the Tx side, which resulted in the alignment of the outcomes of both methods. Figure 17B illustrates a plot for two different distances between the resonator and the Rx, namely 0.5 cm and 1 cm. When the distance between the Tx and resonator exceeds 6 cm, the inductive link exhibits better PTE performance. This performance improvement stems from the coupling coefficient at this distance, which facilitates better matching to the reflected impedance on the Tx side.

### 6.5. PDL Measurement

**Figure 18.**
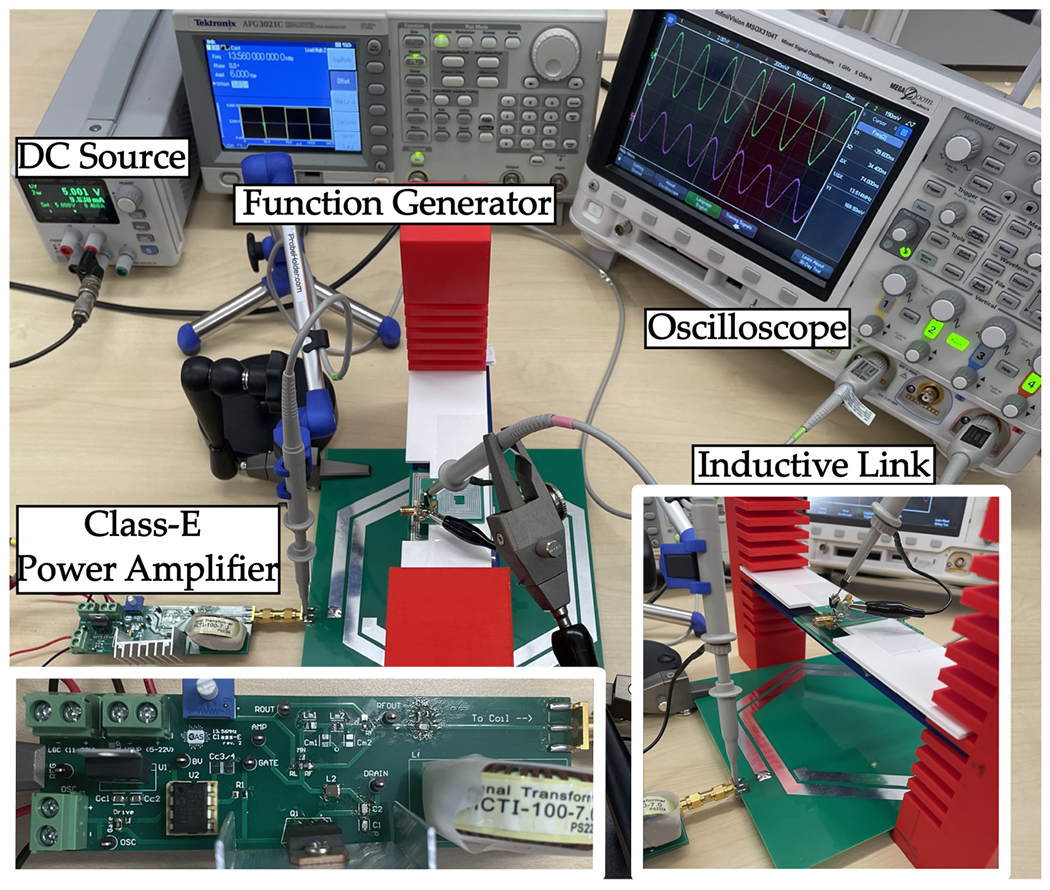
PDL measurement setup.

**Figure 19.**
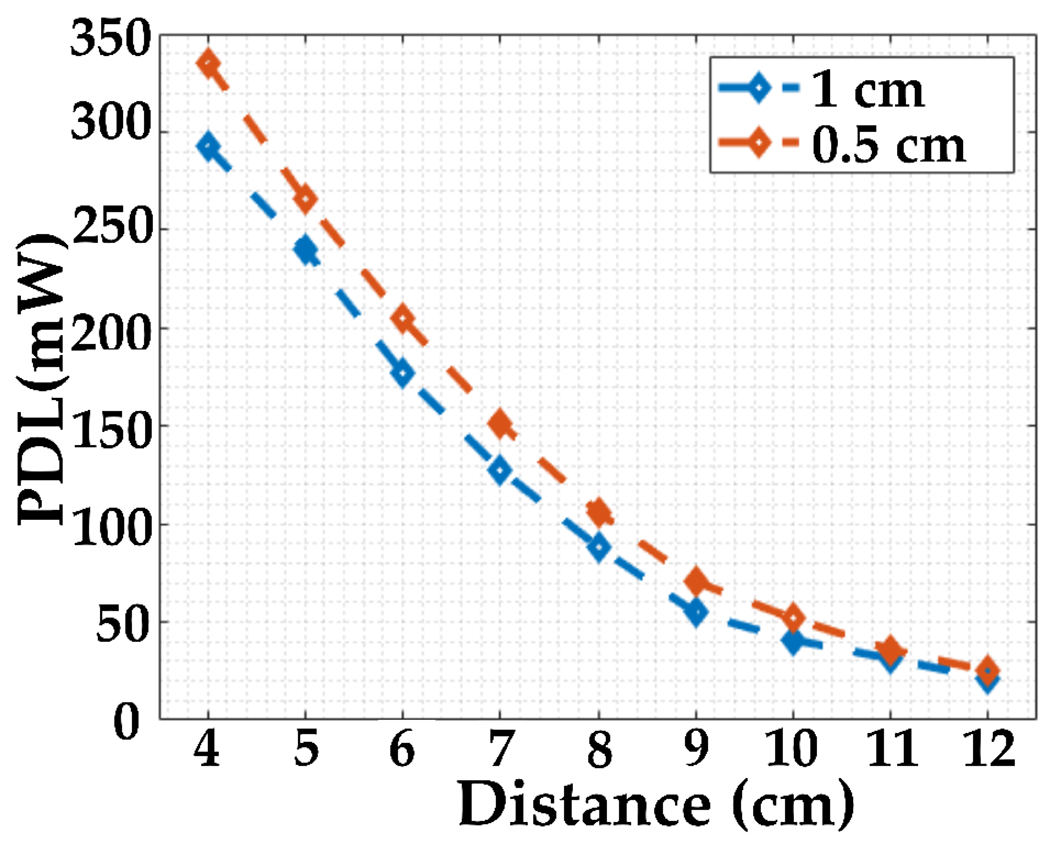
PDL plots for two distances between the resonator and Rx.

To further evaluate the performance of our system, we conducted a series of measurements to analyze the PDL. Figure 18 shows the measurement setup used for these tests. We measured PDL at various distances between the Tx and the resonator, specifically at 0.5 cm and 1 cm separation between the resonator and Rx. The results, plotted in two PDL graphs in Figure 19, indicate that the received power varies from 290 mW at a 4 cm distance to 20 mW at a 12 cm distance between Tx and the resonator. Notably, even in the worst-case scenario, e.g., a distance beyond 12 cm, the received power remains above 10 mW, sufficient to meet the maximum power requirement of the sensor. These findings validate the potential of our wireless power transfer system for NICU sensor applications, providing a robust foundation for further development and implementation.

**Figure 20.**
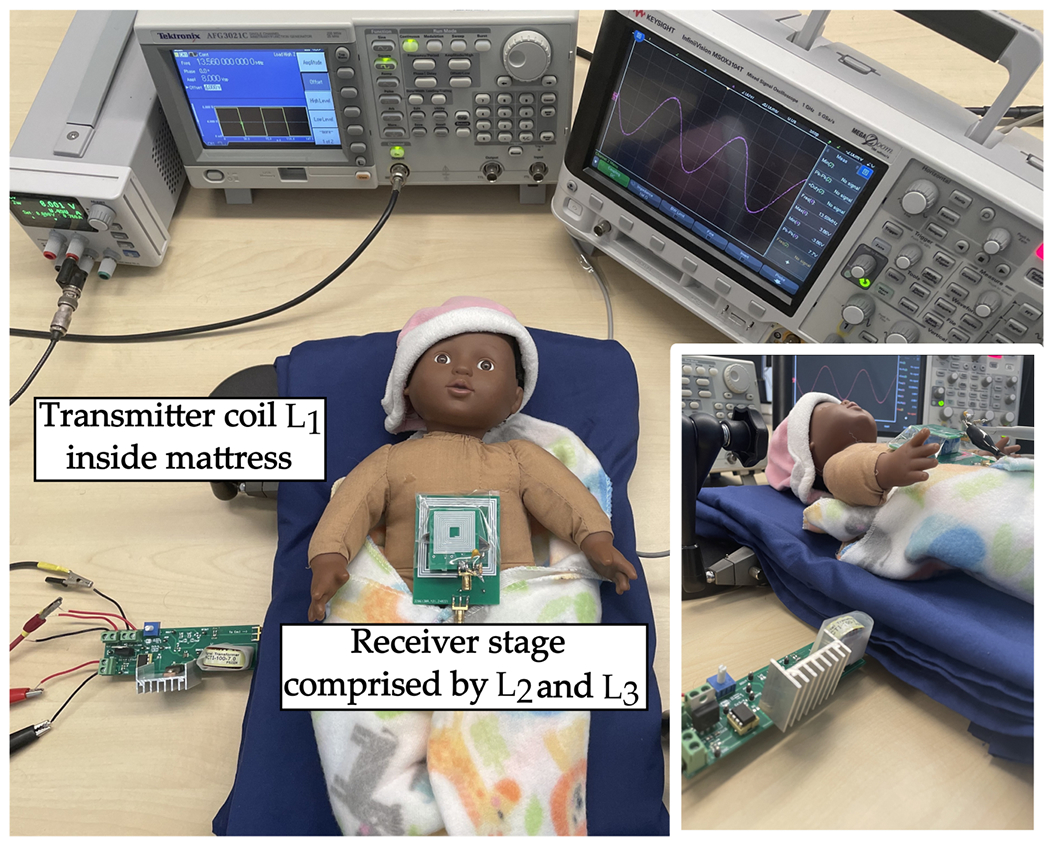
Showcase for the preliminary concept of WPT system in NICU.

The basic concept of our design was investigated where the Tx is integrated into a mattress and connected to a class-E PA. At the same time, both the resonator and Rx are placed on the chest of a dummy baby, illustrated in Figure 20. In this setup, we received 16 mW of power at the 500 Ω load.

We compared the specifications of our inductive link design, designed to be used in a wearable system, with the selected inductive links, designed for various biomedical applications, from the literature in Table 4. Refs. [3] and [90] discuss a two-coil inductive link topology for implantable devices with wire-wound coils. The author of [85] describes a three-coil inductive link topology where the resonator coil is positioned equidistantly between the transmitter and receiver. The author of [32] introduces a cage for freely moving animals to maintain constant PDL within a square area for long-term experiments using a four-coil inductive link topology. Lastly, Ref. [91] presents a three-coil inductive link designed to power free-floating distributed implants in neural tissue.

## 7. Conclusions

This paper provides an overview of key concepts related to inductive power transfer systems, including the significance of the quality factor in coil design, the principles governing mutual inductance calculations for multi-coil systems, and the fundamental configurations of inductive links. Building upon this theoretical foundation, the paper presented a detailed case study focused on the design, simulation, and experimental validation of a three-coil inductive link tailored for powering physiological monitoring sensors in NICU applications. Through a systematic approach to coil optimization, the study aims to achieve high PTE and reliable power delivery over varying distances between the Tx and Rx. This approach underscores the potential of inductive power transfer technology to facilitate continuous monitoring solutions in critical care settings. Finally, the concept introduced in this paper will be further developed and modified to incorporate sensors for neonatal care into a comprehensive system.

## Figures and Tables

**Table 1.** Comparison table of class-D PAs in WPT systems.

Parameter	[56]	[58]	[61]	[62]
Implementation	Off-Chip	0.18 μm CMOS	0.35 μm CMOS	0.18 μm CMOS
Frequency (MHz)	13.56	6.78	6.78	13.56
*P_out_* (W)	7.18	1.68–59.36	0.2–2	30
Efficiency (%)	65–95	92–93	87	80

**Table 2.** Comparison table of Class-E PAs in WPT systems.

Parameter	[72]	[67]	[75]	[47]	[76]
Implementation	Off-Chip	Off-Chip	0.18 μm	Simulation	Off-Chip
Frequency (MHz)	6.78	13.56	13.56	13.56	6.78
*P_out_* (W)	1–22	78.28 m	26 m	1	12
Efficiency (%)	80–88	87.2	50	16	70–82

**Table 3.** Coils specifications.

Parameter	Transmitter *L*_1_	Resonator *L*_2_	Receiver *L*_3_
Inductance (μH)	1.2	1.7	3.1
Outer diameter (cm)	16.8	4.9	2.5
Fill factor	0.14	0.11	0.60
Turns	2	4	13
Width (mm)	8.7	1.0	0.5
Spacing (mm)	2.60	0.35	0.25
Quality factor	173	114	69

**Table 4.** Comparison of state-of-the-art inductive link examples.

Parameter	[3]	[90]	[85] ^1^	[32] ^2^	[91]	This Work
Frequency (MHz)	6.78	6.78	6.78	13.56	60	13.56
Link type	2-Coil	2-Coil	3-Coil	4-Coil	3-Coil	3-coil
Implementation	Wire-wounded	Wire-wound	PCB	PCB	Planar ^3^ and wire-wound	PCB
Distance (cm)	1.2	4	20	4	1.8	12 ^4^
Load (Ω)	2.1 K	100	10	100	500	500
Efficiency (%)	73.6	25.7	55	69	2.4	75 ^5^
Power (mW)	40	720	N/A	120	1.3	330

1 Resonator is positioned equidistant from the Tx and Rx.

2 Values for one power surface.

3 Copper foil was used for the planar coils.

4 Distance between Tx and resonator.

5 Efficiency only for the inductive link.

## Data Availability

Data is contained within the article.

## Abbreviations

WPT: Wireless Power Transfer
IoT: Internet of Things
EV: Electric Vehicles
EM: Electromagnetic
ISM: Industrial, Scientific, and Medical
RF: Radio Frequency
NICU: Neonatal Intensive Care Unit
PA: Power Amplifier
PTE: Power Transfer Efficiency
MTMs: Metamaterials
SAR: Specific Absorption Rate
PMIC: Power Management Integrated Circuit
BJT: Bipolar Junction Transistor
MOSFET: Metal-Oxide-Semiconductor Field-Effect Transistor
ZVS: Zero-Voltage Switching
DDTC: Dynamic Dead-Time Control
DT: Dead-Time
DCM: Discontinuous Conduction Mode
EMF: Electromotive Force
PSC: Printed Spiral Coils
ESR: Equivalent Series Resistance
AME: Additive Manufacturing Electronic
PDL: Power Delivered to Load
AP: Anteroposterior
VNA: Vector Network Analyzer
SMD: Surface Mount Device
